# Magnetic Properties of FeNi/Cu-Based Lithographic Rectangular Multilayered Elements for Magnetoimpedance Applications

**DOI:** 10.3390/s23136165

**Published:** 2023-07-05

**Authors:** Grigory Yu. Melnikov, Irina G. Vazhenina, Rauf S. Iskhakov, Nikita M. Boev, Sergey V. Komogortsev, Andrey V. Svalov, Galina V. Kurlyandskaya

**Affiliations:** 1 Institute of Natural Sciences and Mathematics, Ural Federal University, 620002 Ekaterinburg, Russia; grisha2207@list.ru (G.Y.M.); andrey.svalov@urfu.ru (A.V.S.); 2 Kirensky Institute of Physics, Federal Research Center KSC SB RAS, 660036 Krasnoyarsk, Russia; irina-vazhenina@mail.ru (I.G.V.); rauf@iph.krasn.ru (R.S.I.); boev@iph.krasn.ru (N.M.B.); komogor@iph.krasn.ru (S.V.K.); 3 School of Space and Information Technology, Siberian Federal University, 660041 Krasnoyarsk, Russia; 4 Applied Physics Department, Reshetnev Siberian State University of Science and Technology, 660037 Krasnoyarsk, Russia

**Keywords:** magnetic multilayers, permalloy, magnetic properties, ferromagnetic resonance, spin-wave resonance, magnetoimpedance, magnetic field sensors

## Abstract

The rectangular elements in magnetoimpedance (MI) configuration with a specific nanocomposite laminated structure based on FeNi and Cu layers were prepared by lift-off lithographic process. The properties of such elements are controlled by their shape, the anisotropy induced during the deposition, and by effects associated with the composite structure. The characterizations of static and dynamic properties, including MI measurements, show that these elements are promising for sensor applications. We have shown that competition between the shape anisotropy and the in-plane induced anisotropy of the element material is worth taking into account in order to understand the magnetic behavior of multilayered rectangular stripes. A possibility of the dynamic methods (ferromagnetic and spin-wave resonance) to describe laminated planar elements having a non-periodic modulation of both structure and magnetic parameters of a system is demonstrated. We show that the multilayered structure, which was originally designed to prevent the development of a “transcritical” state in magnetic layers and to reach the required thickness, also induces the effects that hinder the achievement of the goal, namely an increase in the perpendicular magnetic anisotropy energy.

## 1. Introduction

Magnetic film sensors using the giant magnetoimpedance effect (MI) show very high sensitivity within fields of several Oersted. Their effective improvement is closely connected with the development of the deposition technology of the sensitive element and its advanced characterization. Magnetically soft thin films and multilayered structures are widely used in different types of magnetoelectronic applications [1,2]. The evolution of the devices and components with thin-film elements is strongly supported by the well-developed technologies of vacuum deposition and the patterning of different types of thin film media [3,4]. Recently, special attention has been paid to the development of flat sensitive elements for small magnetic field detection at the external field level, which is comparable with the biogenic signals of living systems and the magnetic label detection adaptable for demanding bioanalytic requests [5,6,7,8]. Although there are different magnetic phenomena capable of supporting small magnetic field sensor designs adaptable for particular applications, magnetoimpedance effect (MI) shows very high sensitivity with respect to its applied field in a low field interval [9,10,11].

The MI phenomena is a large variation of the total electrical impedance (Z) of a ferromagnetic conductor under the application of a constant external magnetic field [12,13]. The highest MI effect appears in the case of ferromagnets with well-defined magnetic anisotropy having high transverse dynamic magnetic permeability (μ) [12,13]. It was understood in the frame of a classic electrodynamics approach as a skin-depth (δ) connection with a value of μ: δ = (πfσμ)^−^^0.5^, where f is the alternating driving current frequency, and σ is the conductivity of the ferromagnetic material. This means that for any given material, the MI effect and MI sensitivity (the change per unit of the magnetic field) depends on the frequency of the flowing current and dynamic magnetic permeability.

For a sensor application, the lower working frequency is usually an advantage. A high sensitivity of MI for a frequency at the order of tens of MHz would therefore appear for the thickness (L) of the magnetic layers of the MI element at about 1 micron [14,15,16]. For the deposition of such thick films of suitable soft magnetic materials, some obstacles should be overcome. One of the most used material in sensor applications is permalloy Fe_20_Ni_80_ [17,18,19]. However, aside from the critical thickness (L_c_) at the order of hundreds of nanometers, FeNi films may experience the transition into the “transcritical” state characterized by: an increase in the coercivity H_c_ and particular features of the magnetic hysteresis loop shape; the stripe magnetic domains’ formation; and the appearance of “rotational anisotropy” [20,21,22]. The critical thickness value at which the abovementioned transition takes place depends on the technological parameters of deposition (working gas pressure, the substrate orientation, deposition rate, etc.). It usually appears in the interval of 100 to 350 nm thicknesses [19,20,21,22]. In order to solve this problem, the concept of nanostructuring was proposed [23,24].

**Figure 1:**
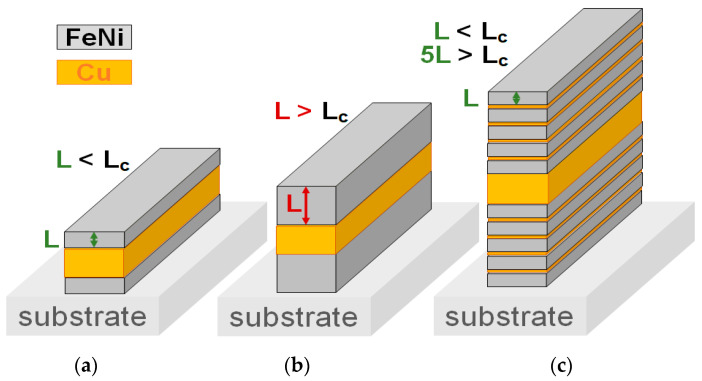
Scheme of rectangular MI multilayered element with open magnetic flux. FeNi top and bottom layers with the thickness below the critical thickness of the transition into a “transcritical” state (**a**). FeNi top and bottom layers with the thickness above the critical thickness (**b**). FeNi top and bottom magnetic layers form the multilayered structure due to nanostructuring by the Cu spacers with the thickness below the critical thickness of the transition into the “transcritical” state for all magnetic sub-layers of FeNi (**c**).

Figure 1 presents an example of the nanostructuring approach. It shows rectangular MI structures with an open magnetic flux: ferromagnet/conductor/ferromagnet structure with the same width and length. In order to obtain a pronounced skin-effect and high MI sensitivity at reasonably low frequencies below GHz intervals, the thickness of FeNi top and bottom layers and Cu central lead should be close to each other, and the thickness of the central lead have to be close to 0.5 μm [9,25]. However, as mentioned before in many sputtering devices L_c_ < 0.2 μm (Figure 1a), a further L increase would cause the transition into the “transcritical” state for top and bottom magnetic layers (Figure 1b).

We are therefore faced with a clear contradiction: for a high MI value, the magnetic softness and thickness of about 0.5 μm are necessary, but thin permalloy films above 0.2 μm lose their magnetic softness due to the transition into a “transcritical” state for both top and bottom magnetic layers. The solution of the abovementioned problem is the usage of a multilayered structure. Figure 1c shows the structure of an MI element with five top and five bottom FeNi layers separated by Cu spacers. Despite the high total thickness 5L > L_c_, the transition into the “transcritical” state does not take place in the multilayered structure. In this case, the value of coercivity H_c_ in a single layer with L < L_c_ and H_c_ in the multilayered structure are close to each over.

Multilayered structures were used in electronic devices long ago [26,27], but nowadays, very complex structures combining magnetic, conductive, semiconductive, and patterned layered components are widely used [28,29]. In the case of MI, two points should be outlined: the need to obtain a particular shape of the magnetic sensitive element due to a strong contribution of shape anisotropy, and a relatively large thickness of the magnetic layers and Cu conductive lead. For the symmetric MI structures, the theories provide the answer for the highest MI effect for FeNi top and bottom layers and Cu central lead, which are close to each other [10,25]. However, the obtained experimental values are still much lower in comparison with the predictions [10]. One of the possible reasons of the smaller MI values in real samples may be a result of the asymmetry of the magnetic properties of an MI multilayered structure’s layers (Figure 1c) due to long deposition time and the presence of a thick Cu layer in the middle. Previously, we have shown [30] that a 0.5 μm thick Cu lead has a rather high average grain size of up to 50 nm in comparison with a typical size of 12–25 nm for thin FeNi films of 100 nm. The grain size and texture of the Cu lead usually contribute to the structural peculiarities of the FeNi layer immediately above the Cu lead, and consequently to the properties of the other layers of the top multilayered structure. As a result, the structure and magnetic properties of the top and bottom multilayers differ from each other and their asymmetrical properties become reflected in MI decrease.

In addition, interfaces in multilayers are sources of the internal deformations, which should result in a shift of the critical thickness due to the magnetoelastic contribution to the perpendicular magnetic anisotropy of the films, as well as due to the influence of the interface to the layer growth mode. For single permalloy layers prepared by magnetron sputtering, it was shown that the constant of perpendicular magnetic anisotropy is the thickness invariant [31]. In a multilayered structure, the numerous interfaces may result in an increase in the value of the perpendicular magnetic anisotropy energy. However, detailed studies regarding this problem are absent in the literature. Thus, to solve it, one could use other well-known microwave techniques, which involve dynamic magnetic permeability: ferromagnetic resonance (FMR) and spin-wave resonances [32]. FMR is a resonant absorption of microwave radiation by magnetic material in a constant magnetic field [33,34]. In the very first years of MI studies, Yelon et al. [31] pointed out that a theoretically calculated MI for a rectangular plate is equivalent to the FMR response of the same plate in the case for which an electric field is constant along the length of the long side of the rectangle. They suggested the procedure to apply all solutions of FMR behavior to the description of MI.

MI sensitive elements described in the literature were usually deposited by sputtering technique using metallic masks or afterward through standard lithography; at times, a micromachining technique was employed [35,36,37]. Lithography is well established and a highly productive technique, and at times, is rather flexible for the particular need of present-day electronics. With this respect, lift-off was shown to be the most convenient method for multilayers with Cu spacers. It allows us to obtain large batches of the sensitive elements at a time, ensuring their identical properties.

Here, we employ ferromagnetic resonance together with studies of magnetic properties and MI for FeN/Cu-based multilayered MI rectangular elements obtained in batches of twelve separate elements by standard lift-off lithographic process in order to understand the high frequency of their properties in detail.

## 2. Experiment

Taking into account the results obtained in previous works [14,23], the magnetic layers of FeNi before and after the central Cu–lead was nanostructured by Cu spacers with the aim to avoid the transition into a “transcritical” state. Despite the fact that Ti nanostructuring seems to be more efficient for MI elements, titanium is not very appropriate for simple lithographic steps as it requires elevated temperatures for chemical processing during lithography. It was mentioned above that a thick Cu layer, which is necessary in MI sensitive elements as a current conductor, has the grain size of the order of 50 nm for a layer of about 500 nm [32]. Deposition of the permalloy layer onto Cu–lead with the grain size of the order of 500 nm inevitably results in an inherited increase in the FeNi grain side and worse magnetic properties developing asymmetry.

We therefore designed a multilayered structure in which the central Cu–lead was also nanostructured. In order to decrease the grain size of the Cu and consequently the thick FeNi layer grown onto the thick Cu layer instead of the one-layered Cu–lead, the multilayered [Cu(3 nm)/FeNi(100 nm)]_5_/Cu(150 nm)/FeNi(3 nm)/Cu(150 nm)/FeNi(3 nm)/Cu(150 nm)/[FeNi(100 nm)/Cu(3 nm)]_4_/FeNi(100 nm) structure was deposited. In order to keep the design as simple as possible, FeNi sub-layers were used for Cu–lead nanostructuring and a whole MI element technological request was limited by only two sputtering targets. Thus, the proposed material corresponds to the concept of technological “minimalism” and can be considered as the basis for a working prototype of a sensor element oriented toward mass technologies.

FeNi/Cu-based multilayered films were deposited by dc magnetron sputtering onto corning glass substrates at room temperature. Corning glass is a widely available amorphous material, which is widely used for thin films deposition, including MI structures [3,5,9,13]. It allows us to obtain the appropriate (111) texture when depositing onto buffer Cu layer and a magnetic softness of FeNi layers. In addition, the glass substrate is transparent, and therefore, one can study the properties of the top and bottom layers using the magneto-optical Kerr effect technique (separately for top and bottom layers). Metallic circular targets of Fe_20_Ni_80_ or Cu compositions were used. An additional calibration procedure for the definition of the deposition rates was performed for each composition using 100 nm films. Each thin film’s thickness for calibration was measured by the sharp step analyzed with Dektak 150 Stylus Profilometer (Veeco, Somerset, NJ, USA). The following deposition rates were used: 26 nm/min for FeNi layers and 13 nm/min for Cu spacers and the central conductive lead. A total of 100 nm thin film of a known area (3.5 mm × 3.5 mm) was used for saturation magnetization (M_s_) definition using magnetic measurements with vibrating sample magnetometer (VSM).

**Figure 2:**
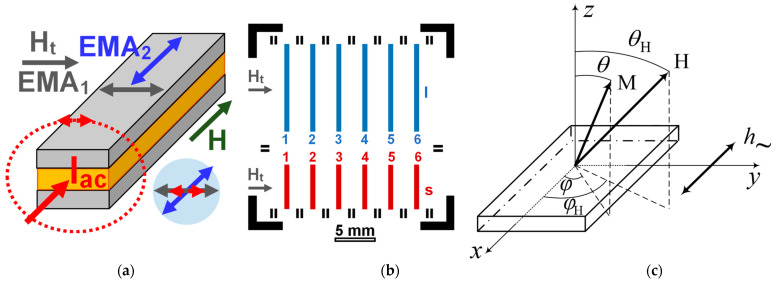
General description of the magnetic fields and magnetic anisotropy contributions. EMA1—easy magnetization axis induced due to deposition in the external field H_t_. EMA2—Easy magnetization axis corresponding to the shape anisotropy. Iac—Direction of the flowing alternating current; red double arrow shows orientation of the magnetic field created by the flowing current (**a**). Scheme of [Cu(3 nm)/FeNi(100 nm)]_5_/Cu(150 nm)/FeNi(3 nm)/Cu(150 nm)/FeNi(3 nm)/Cu(150 nm)/[FeNi(100 nm)/Cu(3 nm)]_4_/FeNi(100 nm) multilayered element in magnetoimpedance geometry. H_t_—Direction of the application of technological magnetic field during deposition. Iac—Direction of the flow of the high frequency alternating current during magnetoimpedance applications. Note that the structures are shown not in their real scale (**b**). Schematic description of the lithographic arrangement of the long (l—blue-colored) and short (s—red-colored) rectangular elements (**b**). The experimental geometry for ferromagnetic resonance measurements. Here, M is the magnetization vector, H is an external constant magnetic field, *h*_~_ is microwave rf-field. For definition of all angles see also the main text (**c**).

As the first step, [Cu(3 nm)/FeNi(100 nm)]_5_/Cu(150 nm)/FeNi(3 nm)/Cu(150 nm)/FeNi(3 nm)/Cu(150 nm)/[FeNi(100 nm)/Cu(3 nm)]_4_/FeNi(100 nm) multilayered films were deposited onto the whole glass substrate with a background pressure of 3 × 10^−7^ mbar and a working Ar pressure of 3.8 × 10^−3^ mbar. Figure 2a describes the multilayered structure. As the presence of the Cu buffer layer for FeNi deposition onto glass substrates results in higher magnetic properties, the Cu buffer layer was deposited first.

For fabrication, a batch of the magnetoimpedance sensitive elements with two different lengths (l for 0.5 mm × 10.0 mm and s for 0.5 mm × 5.0 mm elements) of a standard optical lift off lithography was employed [38]. Figure 2b shows arrangements of the MI elements onto one glass substrate: one batch for fabrication in the same conditions simultaneously. Due to the selected fabrication technique, the obtained magnetoimpedance elements were configurated with open magnetic flux [3,9], i.e., the rectangular elements consisted of a number of layers of the same width and length (0.5 mm × 10.0 mm for l; and 0.5 mm × 5.0 mm for s samples). Two batches of 12 elements (batch I and batch II) were arbitrarily selected for characterization by different techniques. Therefore, the denomination of the elements included the batch number (I or II), the length (s or l), and position (from 1 to 6). Figure 2b shows the arrangement of l and s elements related to one batch of the samples as well as some additional metallic parts, which are used for positioning the elements. For simplicity, the MI elements of l types and their corresponding numbers are represented in blue, and the MI elements of s types and their corresponding numbers are represented in red. All other structures and writings represent scale, external magnetic field orientation during the film deposition, and technological lines for positioning.

During the deposition process, an in-plane constant technological magnetic field H_t_ = 100 Oe was applied along the short side of the MI elements of both lengths in order to induce a transverse uniaxial in-plane magnetic anisotropy. That is, the direction parallel to the short side of the rectangular stripe along which the technological magnetic field was oriented during film fabrication was favorable from the point of view which induced magnetic anisotropy, creating an easy magnetization axis (EMA1). Induced anisotropy, however, competed with the shape anisotropy, for which an easy magnetization axis (EMA2) should be oriented along the long side of the stripe in the plane of the multilayered structure.

Magnetic measurements were carried out by means of 7407 VSM vibrating-sample magnetometer (Lake Shore Cryotronics, London, UK), ferrometer (Laboratory of Scientific Instrumentation of the Institute of Physics SB RAS, Krasnoyarsk, Russia) [38], and magneto-optical Kerr effect (MOKE) using the optical microscope Evico (Evico, Dresden, Germany). The last equipment was also used for the magnetic domain structure observation in different external magnetic fields.

A rectangular multilayered MI sensitive element was placed into a “microstripe” line being contacted by a highly conductive silver painting. A uniform constant external magnetic field (H) of up to 100 Oe was created by a pair of Helmholtz coils. It was applied along the long side of the rectangular element and, therefore, the longitudinal magnetoimpedance configuration was employed: the alternating current was flowing parallel to the external magnetic field, providing the highest sensitivity of the MI ratio. The S_11_ reflection coefficient was measured by a network analyzer (Agilent E8358A) with an output power of 0 dB, corresponding to the amplitude of about 1 mA of the excitation current across the multilayered element. The calibration and mathematical subtraction procedures for the test fixture contributions were performed in accordance with a well-described procedure [39]. Total impedance variation was extracted as a function of the external magnetic field from the variations of the S_11_ reflection coefficient in a frequency range of 0.1–400 MHz. The error in determining the impedance was within 1%. The MI ratio (ΔZ/Z) and MI ratio sensitivity (s(ΔZ/Z)) were calculated as follows:

(1) $$\Delta Z/Z(H)=\frac{Z(H)-Z(H_{max})}{Z(H_{max})}\cdot 100\%$$

where *Z*(H) and Z(H_max_) are the impedance moduli corresponding to the external magnetic fields H and H_max_, respectively. The magnetic field sensitivity of the MI ratio, i.e., the change of the MI ratio per unit of the external magnetic field was determined by the following expression:

s(∆Z/Z) = Δ(∆Z/Z)/∆H
(2)

where ΔH = 0.1 Oe—increment for an external magnetic field.

The ferromagnetic resonance of the multilayered structures was studied on the basis of the measurements of absorption spectra by a cavity perturbation technique using a standard Bruker spectrometer (Elexsys E580, Bruker, Germany) at room temperature at a pumping frequency of 9.4 GHz (X-band) and at the transverse pumping of the cavity. The sample was placed in an antinode of a variable magnetic radiofrequency field (*h*_~_) for conventional homodyne detection and rectangular cavity. The microwave absorption curves were measured as a function of the applied magnetic field. The experimental curves were decomposed into components using the differentiated Lorenz function chosen by taking into account the absence of the contribution of the microwave electric part of the field. The last was possible due to the cavity construction and sample size. The measurements were carried out with the direction of the constant magnetic field *H* changing both in the plane which is parallel to the film normal (the angle $\theta_{H}$ is variable) and in the film plane (the angle $\phi_{H}$ is variable) (Figure 2c). In all configurations, the radiofrequency magnetic field was perpendicular to the external constant magnetic field: *h_~_*⊥ *H*.

## 3. Results and Discussion

### 3.1. Static Magnetic Properties

**Figure 3:**
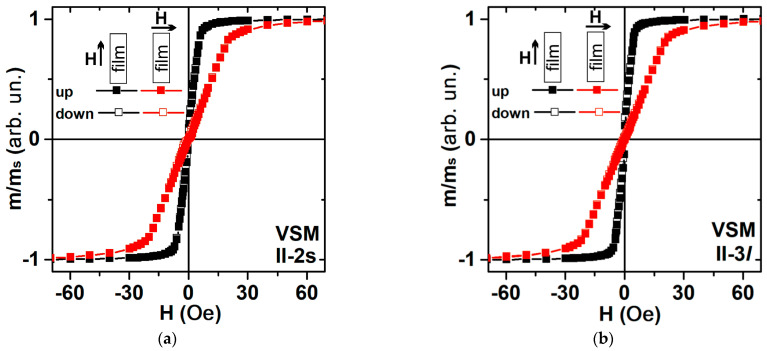
VSM in-plane magnetic hysteresis loops measured along the long and along the short sides of magnetoimpedance elements: short rectangular MI element II-2s (**a**); long rectangular MI element II-3*l* (**b**).

**Figure 4:**
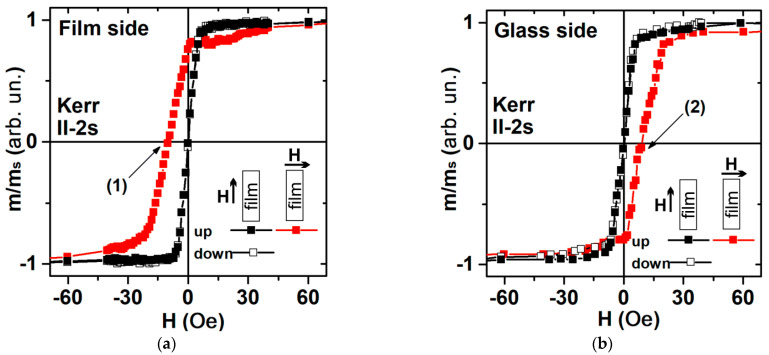
MOKE magnetic hysteresis loops for short rectangular element magnetoimpedance element II-2s measured along long and short sides of element from the side of the film (top layer) (**a**); from the glass side: (**b**). The numbers (1) and (2) indicate the hysteresis loop points, for which the images of magnetic domains were collected (see also Figure 5).

Figure 3 shows representative examples of magnetic hysteresis loops obtained using VSM and Figure 4 represents magneto-optical Kerr effect data. The external magnetic field H is applied parallel to the plane of the substrate and in two directions perpendicular to each other: one parallel to the long side of the multilayered rectangle depicted in Figure 2a, and one perpendicular to the short side of the multilayer, i.e., parallel to the H_t_ technological field also shown in Figure 2a. The VSM data are associated with the response of the whole sample. However, in the case of the MOKE technique, it is related to the surface layer of about 20 nm in FeNi alloys [40,41].

Figure 2a describes possible contributions to the effective magnetic anisotropy: induced magnetic anisotropy and shape anisotropy. According to the shape of the VSM hysteresis loop, the easy magnetization axis for the effective anisotropy (EMA) is oriented along the long side of the element, the coercive force is about H_c_ ≈ 1 Oe, and the magnetic saturation field H_s_ = 7 Oe (Figure 3, black curve). The red curve (Figure 3a) describes the magnetization process for the external magnetic field applied along the short side of the rectangular stripe: technical saturation is observed in fields of the order of 30 Oe, which is the result of a mutual contribution from the effective shape magnetic anisotropy and induced magnetic anisotropy of the film (see discussion in Section 3.2).

MOKE hysteresis loops were measured for the upper (free film) and lower (glass contacted) layers of the film structure. According to the results of Kerr microscopy in the surface layer, the magnetic anisotropy axis lies along the short side of the samples and it is governed by the induced magnetic anisotropy. An interesting feature is that the re-magnetization of the upper layer of the multilayered film structure begins in a field directed along the current magnetization vector [42] (Figure 4a, black curve).

The magnetization reversal of the lower layer (adjacent to the glass) begins as the classical process of magnetization reversal: it starts when the external magnetic field vector is directed against the current magnetization vector (Figure 4b, red curve). The magnetization reversal process occurs by the displacement of the magnetic domain walls for the orientation of the external magnetic field along the short side of the rectangle stripe (Figure 4a,b).

**Figure 5:**
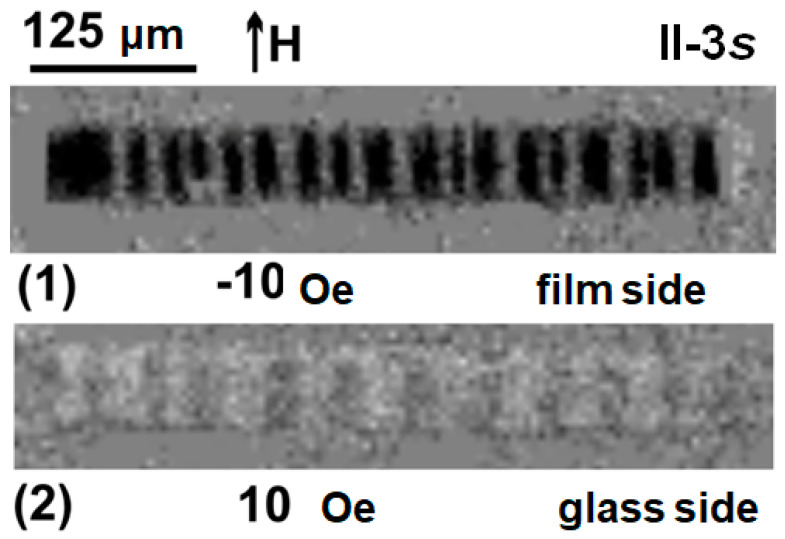
MOKE magnetic domain structures of whole surface of the 2s element observed from the side of the film (1) and from the side of the glass (2). The images were taken near the coercive field (see also Figure 4).

**Figure 6:**
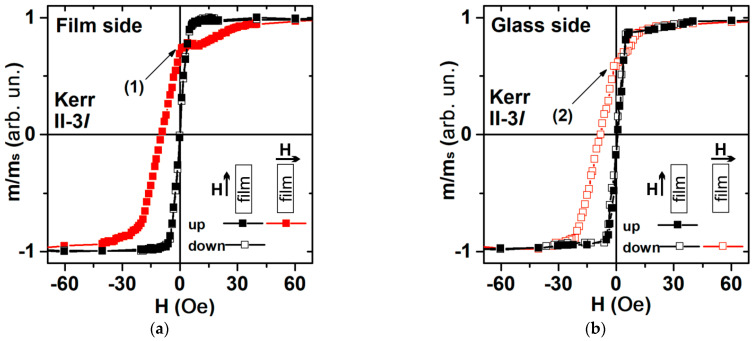
MOKE magnetic hysteresis loops for element II-3*l*: from the side of the film (top layer): (**a**) along the short side from the glass substrate side: (**b**) along the short side of the element. The numbers (1) and (2) indicate the hysteresis loop points, for which the images of magnetic domains were collected (see also Figure 7).

When the external constant magnetic field is directed along the long side of the MI element, the magnetization reversal occurs due to the rotation of the spontaneous magnetization vector. The magnetic anisotropy field was of the order of H_a_ = 7 Oe, which coincides with the VSM measurement results (Figure 4 and Figure 6 black curves). It is worth mentioning the good agreement between magnetic measurements and the magnetic domains observations. The latter confirms that the technological magnetic field induced a transverse magnetic anisotropy in the rectangular element: the orientation of the 180° domain walls is parallel to the short size of the rectangle and the average size of magnetic domains of both phases, which is close to 0.25 mm (Figure 5). The main features of magnetization reversal processes are similar for long and short samples (Figure 4 and Figure 6).

The magnetic hysteresis loops measured along the short side of the rectangular element for the top layer and the layer from the transparent glass substrate side repeat each other. The M(H) have a slope in the field range from remanence to saturation magnetization (Figure 6, red curves). It is associated with the magnetization reversal of individual areas of the rectangular element.

As for the case of the sample II-2s, 3250-3l element has a coercive force H_c_ = 10 Oe according to Kerr microscopy. However, according to the results of VSM magnetometry, the coercive force is about 1 Oe. In this case, the Kerr microscopy method determines not the coercive force, but the field of magnetostatic interaction, which is created by layers in the depth of the element on the surface layers and vice versa. The transverse induced magnetic anisotropy also makes a significant contribution to the effective magnetic anisotropy of the long samples. One can also notice that the orientation of the 180° domain walls is parallel to the short size of the rectangle element.

Based on the fact that in a zero field the lower and upper layers differ in the direction of the magnetization, it can be assumed how the magnetization is distributed in individual layers inside the rectangle MI sensitive element. Apparently, taking into account the number of FeNi layers, the direction of magnetization can be constructed following a schematic representation of the magnetic moments’ re-arrangements (Figure 8). Thus, nanostructuring with the primary purpose to avoid the transition into a “transcritical” state is indeed a very complex process affecting different contributions to the total energy balance.

### 3.2. Magnetic Anisotropy of Rectangular Lithographic MI Elements

The most significant contribution to the in-plane magnetic anisotropy of thin magnetic film elements is the magnetic anisotropy of the shape. Thus, Figure 9a shows that magnetizing along the rectangular stripe element axis requires smaller external fields than in the element transverse direction. The observed magnetic saturation field is correlated to the demagnetizing field *H_d_*. In a laminar composite film element, according to an effective media approach [43,44,45], this field can be described as follows:

(3) $$H_{d}=M_{s}\cdot f\cdot \left({f\cdot N}_{s}+{(1-f)\cdot N}_{p}\right)$$

where *N_s_* is the effective demagnetization factor of the whole sample (assuming that the magnetization is uniform within it); *N_p_* is the demagnetization factor of the parts that make up the laminar film composite; and *f* is the volume fraction of the magnetic component in the composite film consisting of magnetic layers and non-magnetic spacers.

To evaluate *H_d_* using Equation (1), we took *M_s_* = 780 G for permalloy and *f* = 0.67. The *N_s_* and *N_p_* values were calculated using the equation for a rectangular prism [46]: for *N_s_*, the prism parameters were taken equal to the sample sizes (a = 10 mm for the long and 5 mm for the short, b = 0.5 mm and c = 1.5 µm) and for *N_p_*, the layer parameters above and below the central copper layer were used (a =10 mm for long and 5 mm for short, b = 0.5 mm and c = 0.5 µm). Each of these two layers is also a composite; however, this can be neglected in the calculation due to the smallness of the contribution from the thinnest layers (100 nm).

The estimates of the demagnetizing field values (Table 1) are somewhat higher than the saturation fields *H_s_* observed in the experiment (measured in the way shown in Figure 10b) for a field orientation transverse along the long axis of the element, but it is lower than *H_s_* for field orientation along the element’s long axis. This is due to the fact that the film material itself is characterized by the easy magnetization axis induced during the deposition in an external magnetic field. This anisotropy has been studied in detail for similar types of films [34]: its easy magnetization axis is along the direction of the external magnetic field applied during element deposition, and the field value of such a uniaxial anisotropy *H_ip_* is about several Oe, which is quite reasonable for many sensor applications.

Since the field was applied across the long axis of the element during the deposition of the elements under study, the easy magnetization axis of this induced anisotropy is perpendicular to the easy magnetization axis associated with the shape anisotropy. The competition between these two contributions at different orientations of the in-plane field makes the observed differences between *H_d_* and *H_s_* in Table 1 clear.

**Figure 7:**
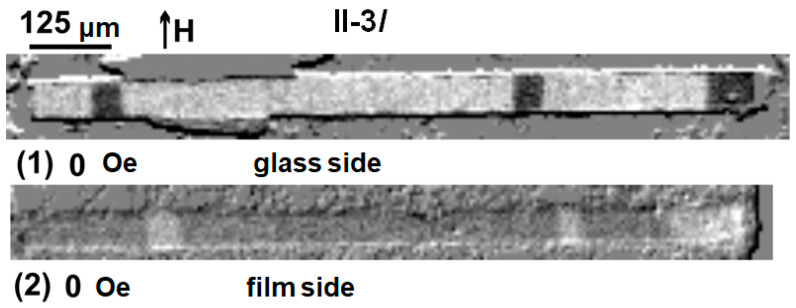
MOKE magnetic domain structures observed on whole surface of the II-3*l* element studied both from the side of the film (1) and from the side of the glass substrate (2). The images were taken near the coercive field (see also Figure 6).

For the field orientated along the long side of the rectangular element, the main contribution to the saturation field *H_s_* comes from H_ip_, while the contribution from the field *H_d_* increases *H_s_*, as is expected from the Stoner–Wohlfarth model, when two uniaxial contributions with an orthogonal orientation of the easy magnetization axis compete. For the transverse orientation, the main contribution to the saturation field *H_s_* is *H_d_*, while the field *H_ip_*, on the contrary, reduces *H_s_*. Close to the demagnetized state of the element, the induced anisotropy with the easy magnetization axis transverse along the long axis of the element remains the only magnetic anisotropy contribution, which is clearly observed in the domain structure of the elements (see Figure 5 and Figure 7).

The coercive fields of the elements (Figure 11a) are when: the field is applied along the element long axis, *H_c_* is within 0.5 ÷ 0.9 Oe, and the elements with a smaller aspect ratio show a smaller *H_c_* value.

The value of M_r_ when the field is applied along the long axis of the element reaches the maximum value M_r_/M_s_ = 0.06. The behavior of M_r_ reducing to this maximum remanence (Figure 10b) provides additional support to the conclusion that the magnetization axis orientation is easy, and it also reveals the remarkable repeatability of this characteristic such as in comparing the properties of the samples with different lengths. Briefly, the angular dependence of the coercivity and the remanent magnetization of the rectangular MI element only reveal and confirm the dominant contribution of the shape anisotropy of such a magnetic composite sample. The magnetic anisotropy of the element determines the magnetic susceptibility, the important characteristic that can be associated with such a sensor parameter as its sensitivity. The field behavior of the differential susceptibility χ estimated as: χ = dM/dH (Figure 11a) shows that in addition to a flat plateau in a certain range of fields near zero, a peak is observed in negative fields.

**Figure 8:**
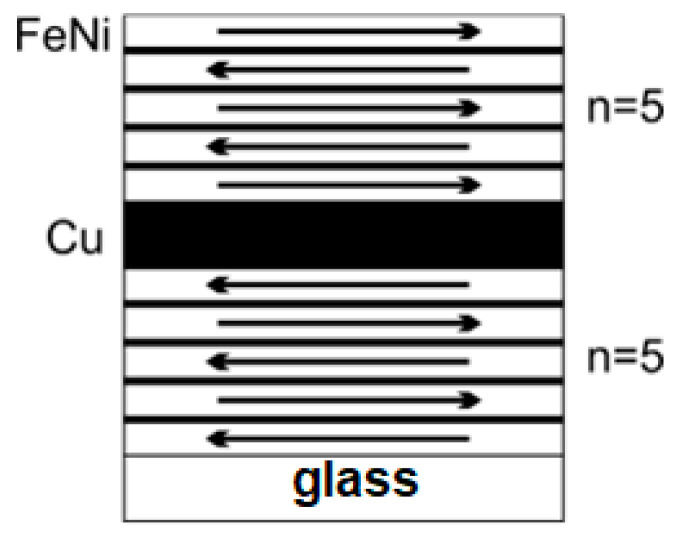
Scheme of the direction of magnetization in the layers of the MI element film structure in a zero field in the demagnetized state.

**Figure 9:**
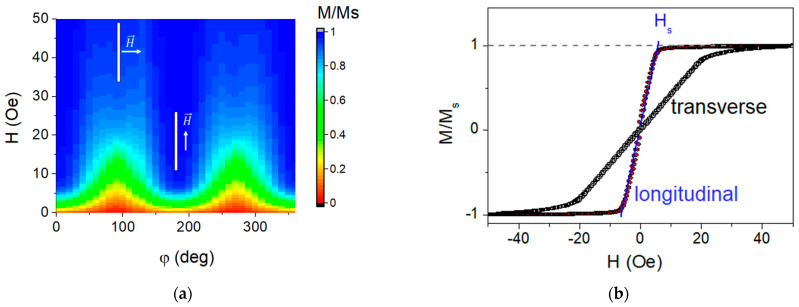
(**a**) Magnetic anisotropy of elements (using example of the element I-3*l*), the angle is measured from the direction of the long axis of the element; (**b**) magnetization curves along and across the long axis of the element.

**Figure 10:**
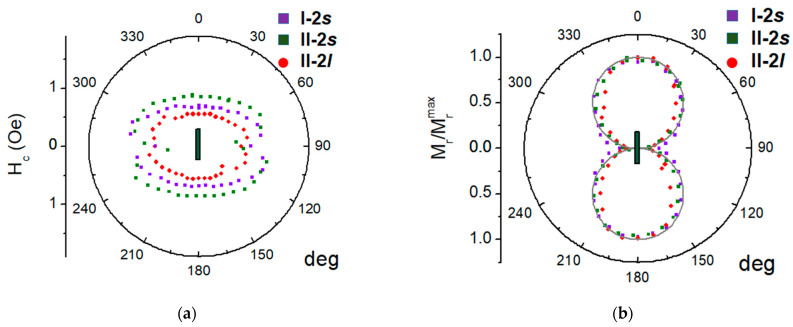
Coercive field (**a**) and the reduced remanent magnetization; (**b**) of the 3 different elements, the green rectangle shows the element orientation, solid line is the cos(φ).

**Figure 11:**
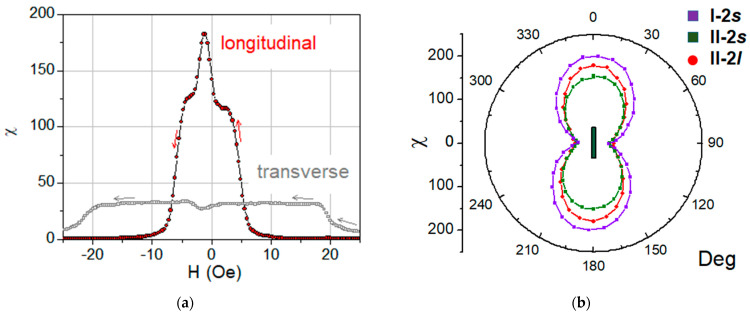
(**a**) Magnetic susceptibility of element l (only the descending branch of the field dependence is shown, the ascending branch behaves symmetrically about the vertical axis), (**b**) the anisotropy of the maximal magnetic susceptibility where the angle is measured from the long axis of the MI element.

This peak, which is related to a sharp drop in the magnetization curve, is most likely due to the formation and mobility of the domain structure shown in Figure 6 and Figure 8, as well as possibly due to the magnetization reversal of the layers in the composite with dipole–dipole interaction between its elements. The anisotropy of the maximum susceptibility (Figure 11b) is qualitatively similar for different elements, although its magnitude is somewhat different.

### 3.3. Selected Examples of Ferromagnetic and Spin-Wave Resonances

**Figure 12:**
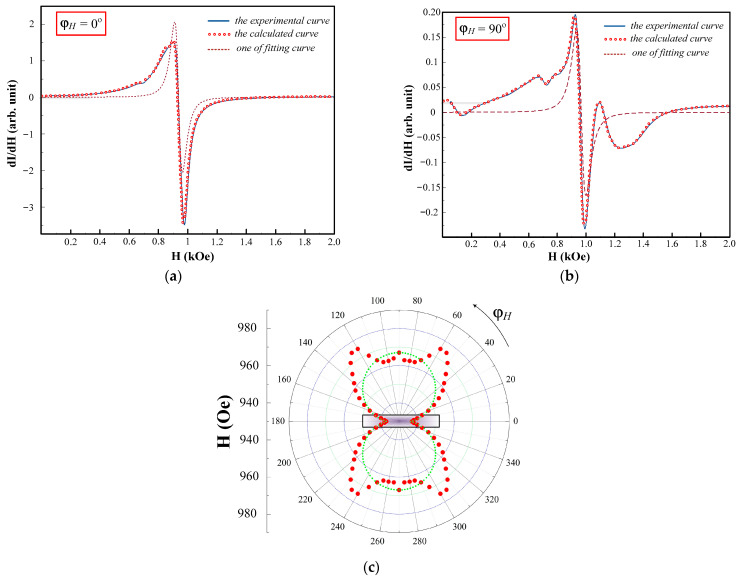
Ferromagnetic resonance spectra at the angles $\phi_{H}=0{}^{\circ}$ (**a**) and $\phi_{H}=90{}^{\circ}$ (**b**) and the angular dependence of selected mode (**c**) for the rectangular MI multilayered II-3s element. The red dots show the experimental values of the resonant field, the green line shows the position of the resonant fields in the case of uniaxial anisotropy.

The microwave spectra of the film measured at the in-plane orientation have complex structures which differ for the varied values of $\varphi_{H}$. The recorded spectra can be divided into two types in terms of complexity, which are illustrative of two different ranges of $\varphi_{H}$. The criteria of the complexity were: the number of Lorentzian functions needed to decompose an experimental curve, as well as the deviation value of a spectrum form from Lorentzian. Examples of spectra for the first ($300{}^{\circ}<\varphi_{H}<60{}^{\circ}$ and $120{}^{\circ}<\varphi_{H}<240{}^{\circ}$) and the second ($60{}^{\circ}<\varphi_{H}<120{}^{\circ}$ and $240{}^{\circ}<\varphi_{H}<300{}^{\circ}$) ranges are represented in Figure 12a,b, respectively.

**Figure 13:**
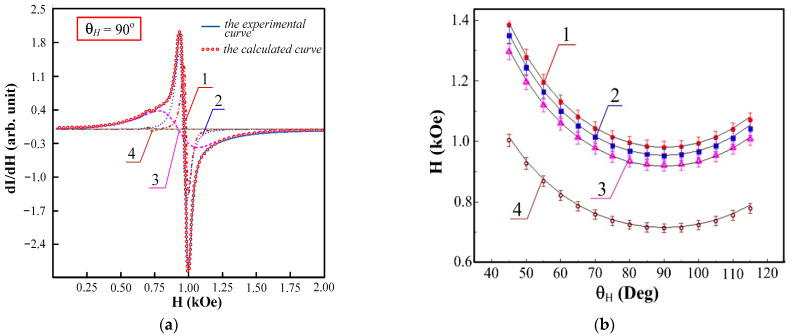
Example of the experimental spectrum at out-of-plane orientation decomposed into components (**a**) and the comparison between the experimental points of the resonance fields and the fitting curves (**b**). Numbers indicate individual fitting Lorentzians.

The deviation value of the spectrum from Δ was evaluated on the basis of a difference between the square of one Lorentzian and the square limited by the experimental curve. $\Delta_{1}$ was about 14 ± 2% for the first range and $\Delta_{2}$ was about 45 ± 5% for the second range. The number of Lorentzian functions needed to decompose the experimental curves were rather large: four for the first range and six to eleven for the second range. The most complex shape of the spectrum was observed for $\varphi_{H}=90{}^{\circ}$. The individual Lorentzian considers corresponding to an excitation of uniform oscillations of magnetization modes in an effective layer, which can be assigned from one to several layers of permalloy. Each individual effective layer is described by its inner field [47]. Figure 13a,b (dashed lines were used for calculated and fitted curves) were used for the construction of the angular dependence of a resonance field $H_{//}(\varphi_{H})$ for the in-plane orientation (Figure 12c). One can notice the existence of several axes of the anisotropy in the plane of the element and an unusual angular dependence behavior in the $\varphi_{H}$ angle range of 60 to 120°.

**Figure 14:**
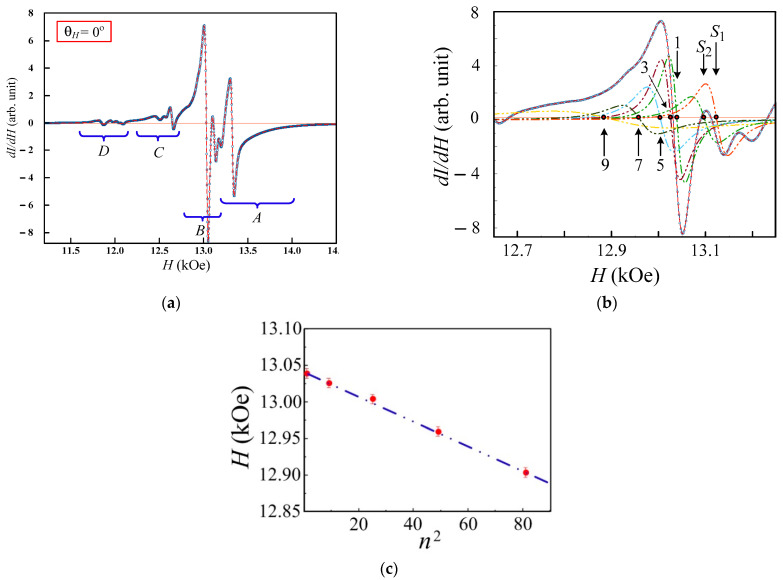
The microwave spectrum at $\theta_{H}=0{}^{\circ}$ (**a**), the example of part B decomposed into components using the differentiated Lorenz function (**b**) and the dependence of the resonance fields of fitting modes on the square of mode number n (**c**). The letters A, B, C, D mark individual parts in the resonance curve, which we refer to individual effective layers of the film. The numbers (1, 2, … 9) indicate individual fitting Lorentzians. The red dots on fragment c show the positions of the resonant fields of part of the spectrum B, the blue line indicates the dependence of the positions of the resonant fields on the square of the mode number.

The microwave absorption spectra of the multilayered film element measured in the out-of-plane orientation also have the complex structure. The experimental spectra in the range of $90{}^{\circ}<\varphi_{H}<40{}^{\circ}$ can be decomposed using four Lorentzian functions (Figure 14a); the angular dependencies of the ferromagnetic resonance fields of which are shown in Figure 13b.

The resonance frequency *ω*_0_ for ferromagnetic resonance [48,49,50] can be described using the total energy of the magnetic system *E* and taking into account the Landau-Lifshitz equation for the motion of magnetization *M*:

(4) $$\omega_{0}=\frac{\gamma }{M\sin\theta }{\left[\frac{\partial^{2}E}{\partial \theta^{2}}\cdot \frac{\partial^{2}E}{\partial \varphi^{2}}-{\left(\frac{\partial^{2}E}{\partial \theta \partial \varphi }\right)}^{2}\right]}^{1/2}$$

where $\gamma =1.758\cdot 10^{7}\;\mathrm{Hz}/\mathrm{Oe}$ is the gyromagnetic ratio, *θ* and *φ* is the polar and azimuthal angles of magnetization in the spherical coordinate system.

In this case, the equilibrium position of the magnetization vector is detected:

(5) $$\frac{\partial E}{\partial \varphi }=\frac{\partial E}{\partial \theta }=0$$

as well as the free-energy density [49]:

(6) $$\begin{array}{l} E=-M\cdot H\left[\sin\left(\theta \right)\cdot \sin\left(\theta_{H}\right)\cdot \cos\left(\varphi -\varphi_{H}\right)+\cos\left(\theta \right)\cdot \cos\left(\theta_{H}\right)\right]+\\ +\left[2\pi M^{2}+K_{n}\right]\cdot \cos^{2}\left(\theta \right)+K_{u}\cdot \sin^{2}\left(\theta \right)\cdot \sin^{2}\left(\varphi -\varphi_{0}\right), \end{array}$$

where *K*_1_ and *K*_2_ are the first and the second cubic anisotropy constants; *K_n_* is the perpendicular uniaxial anisotropy constant; *K_u_* is the in-plane uniaxial anisotropy constant; and *φ*_0_ is an angle describing the direction of the uniaxial anisotropy field in the plane. The contributions of cubic anisotropy are excluded because the film is nanocrystalline.

The angular dependencies of the selected four modes were calculated using the system of Equations (4)–(6) taking into account that the anisotropy field (2*K_u_*/*M_s_*) in the plane of the film is equal to 37 Oe and the effective magnetization is equal to 820 G. In order to obtain a good agreement between the calculated curve and the experimental curve, the value of the perpendicular magnetic anisotropy field *H*_a_ = 2*K_n_*/*Ms* was varied. The calculation was considered to be satisfactory if a difference between the values under consideration was less than 5%.

The values of the anisotropy field H_a_ defined by this method are given in Table 2 and their accuracy was confirmed by the other methods described in the literature [50]. It is necessary to note that the angular range which considered the perpendicular magnetic anisotropy field estimation was limited, and it resulted in the broad range of the possible values of *H_a_*.

In a single permalloy layer, *H_a_* ≈ 130 Oe [31,34] is lower than the centers of confidence intervals given in Table 2. An increase in the perpendicular magnetic anisotropy in a laminar composite is associated with the presence of additional interfaces. It also provides an additional magnetoelastic contribution to the magnetic anisotropy field value. Note that an increase in the magnetic anisotropy field will inevitably lead to a decrease in the critical thickness $L_{\mathrm{cr}}=2\pi \sqrt{\left(A/K_{n}\right)}$. This means that in a composite film element, one has the following dilemma. The concept of nanostructuring sketched in Figure 1 makes it possible to achieve the desired condition L < L_c_. The success of this approach has been established in practice and reported by different authors [5,9,23]. In addition, the fact that the value of L_c_ is the thickness invariant in permalloy monolayers [34] may raise expectations that the simple geometric idea illustrated in Figure 1 would always be sufficient in the case of complex multilayer structures. However, the results of Table 2 indicate that a laminate composite L_c_ will decrease due to the growth of $K_{n}$; thus, the concept of Figure 1 generates conditions that make it difficult to achieve the key condition L < L_c_. From the static magnetic and impedance properties, it follows that the film element is in the subcritical state as a whole, and therefore, the inequality L < L_c_ is valid.

A direct estimate of L_c_ using the data in Table 2 does not contradict this conclusion for the effective layers associated with peaks 1 and 2, although the value of L_c_ is somewhat reduced. Peak 3, apparently, has a composite character. In this regard, it is difficult to consider the parameters of the effective layer associated with this peak as reliable. Peak 4 most likely refers to permalloy layers inside the central copper core layer; its mass is so small that it does not affect the integral responses in any way. As a result, despite the difficulties associated with an increase in the perpendicular magnetic anisotropy in the composite film, an acceptable level of this anisotropy constant is retained, generally leading to the successful preparation of a sensor element with permalloy layers in the subcritical state. This illustrates how closely a successful result is linked to the art of film preparation and deposition techniques.

**Figure 15:**
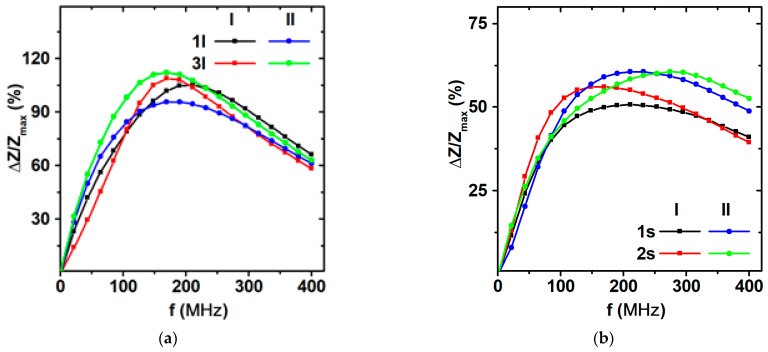
Frequency dependence of the maximum MI ratio for FeNi/Cu-based lithographic rectangular multilayered elements of both studied batches and types (see the legends): (**a**) long elements (l); (**b**) short elements (s).

The experimental spectrum at the out-of-plane orientation ($\theta_{H}=0{}^{\circ}$) has the structure which can be selected on four individual areas such as A, B, C, and D (Figure 14a). The individual areas are described by their sets of standing spin exchange modes (Figure 14b), the resonance fields *H*_n_ of which coincide with a high accuracy of a linear dependence on a squared mode number (Figure 15c). They can be analyzed using the equation [51,52]:

(7) $$H_{n}=\frac{\omega_{0}}{\gamma }+4\pi M_{eff}-\eta_{eff}k^{2}$$

where $\eta_{eff}=2A/M_{S}$ is the spin-wave stiffness associated with the exchange interaction constant A; *M*_s_ is the saturation magnetization; and k=πn/d is the wave vector depending on the mode number n and the film thickness d.

Thus, the measurements at the out-of-plane orientation confirm the conclusion about the existence of several effective layers distributed through the thickness of the planar composite structure, each of which are described by their own set of magnetic parameters (inside field, perpendicular anisotropy field) [53]. The recording of the spin-wave resonance spectrum at the out-of-plane orientation and the linear dependence of the resonance fields on the square of the mode number (Figure 14c) maintains the idea of a homogeneous distribution of the magnetic parameters through the thickness of each effective layer [6]. The last conclusion is based on the results of the studies [54,55,56] that show that a heterogeneity of the magnetic parameters generally results in a different deviation from the low square dispersion.

We would like to mention that each of the presented dependences (Figure 13b and Figure 14c) is a comparison of experimental values (symbols) with values that are calculated from theoretical expressions (lines). The extent to which how well the experimental values related to the numbers of the fitting curves is already a measure of the experimental error. The curves presented in Figure 13b were calculated from the solution of the system of Equations (4)–(6). The fitting straight in Figure 14c follows the linear dependence of resonant fields on the square of the mode number, which was proposed by C. Kittel (Equation (7)) [51]. The experimental spectra were processed using the MagicPlot licensed program (https://magicplot.com/ accessed on 1 January 2023); the fitting error in this program was controlled by the operator and, when analyzing the experimental curves, was no more than 0.5%. In the case of a parallel orientation of the sample (where the resonant fields are in the region of 1000 Oe), the error in determining the field of the fitting curve was no more than + (−) 0.5 Oe, with a perpendicular orientation (resonant fields are 13,000) of 65 Oe. The perpendicular geometry of the experiment was also controlled by the ratio of the intensities between adjacent peaks and should generally decrease with increasing mode number, but it is important to understand that the intensity of the first volume mode is strongly dependent on the surface conditions and thickness of the effective layer within which the standing wave is fixed.

The spin-wave stiffness ${\tilde{\eta }}_{eff}=(H_{1}-H_{n})/(n^{2}-1)$ in the field coordinates and the surface anisotropy constant *K*_s_ were estimated using Equation (7) and presented in Table 3. The difference detected for this laminated film from the value for a single-layer film (${\tilde{\eta }}_{eff}=50\pm 1$ Oe at the film thickness equal to 100 nm) can confirm the suggested model of the effective layer, consisting of alternating ferromagnetic (FeNi) and non-magnetic (Cu) layers. The effective exchange under such a laminated structure is defined by the two contributions: the partial exchange of the ferromagnetic layer and the partial exchange between the ferromagnetic layers via a nonmagnetic interlayer [32,54,55,56,57,58].

The spectra in the angle range of 40 to 10° $\theta_{H}$ are described using modes, a number of which are more than 4. The assumption made above about the existence of various space areas can also be used to conclude that each area has its own boundary conditions and its own angle of transition from a homogeneous type of oscillation to an inhomogeneous one [52]. Therefore, near the angle $\theta_{H}$ equal to 10°, the spectrum can be represented both by homogeneous magnetization fluctuations of one area and by standing exchange spin modes excited in another area. Here, such dynamic methods as ferromagnetic and spin-wave resonances allowed us to describe laminated MI structures having non-periodic modulations of both structure and magnetic parameters of the system. The measurements carried out at in-plane and out-of-plane orientations were useful for the definition of the angular ranges of the orientation of the applied magnetic field, within which the system can be viewed as an effective medium with a small dispersion of average parameters. The analysis of the angular dependences allowed the estimation of a number of fundamental magnetic parameters: the effective magnetization, the exchange interaction constant, the surface anisotropy constant, and the perpendicular anisotropy field.

### 3.4. Magnetoimpedance of FeNi/Cu-Based Lithographic Rectangular Short and Long Multilayered Elements

The magnetoimpedance effect was studied for short and long elements from the I-type (I-1s, I-2s, I-1*l*, I-3*l*) and II-type (II-1s, II-2s, II-1*l*, II-3*l*) series. The maximum MI values (ΔZ/Z_max_) of the magnetoimpedance ratio at different frequencies for l, elements which were approximately twice as high, in comparison with ΔZ/Z_max_ for s elements. The difference is associated with a decrease in the length of the sample [59]. The peak of the frequency dependence ΔZ/Z_max_ for s elements appeared close to about 50%, and for l elements, it was close to about 105% when observed at the driving current frequency of approximately 169 MHz. With a further increase in the frequency, MI ratio decreases. In the case of s elements, the decay was much slower than for l elements (Figure 15). The external magnetic field dependences of the maximum value of MI ratio for l elements show a certain degree of difference from sample to sample, with a minimum of about 93% and a maximum ΔZ/Z ratio of about 105% (Figure 15a). The absolute maximum value of 105% was observed in the external magnetic field of the order of 6 Oe. It is close to the value of the magnetic anisotropy field (7 Oe). The maximum of the sensitivity of 30%/Oe observed in the range of the external fields of 3 Oe to 5 Oe was rather satisfactory for different applications [15,60,61,62].

At the same time, the difference between the maximum value of ΔZ/Z ratio was observed not for different batches, but rather for the elements of the same batch. This might be an indicator of a strong contribution of the surface effects related to the quality of the boarders of lithographic elements obtained by chemical etching. Similar tendencies were revealed for the short samples.

**Figure 16:**
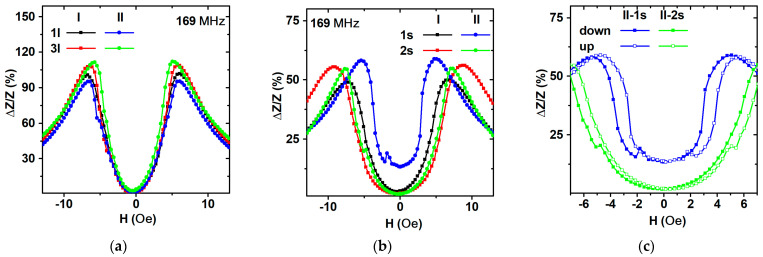
Field dependence of the maximum MI ratio for FeNi/Cu-based lithographic rectangular multilayered elements: (**a**) long elements from batches I and II; (**b**) short elements from batches I and II; (**c**) short elements: “up” is the MI branch measured in increasing and “down” in decreasing external magnetic fields.

The field dependences of the ΔZ/Z ratio for s-type elements were reasonably close to each other in the maximum value ΔZ/Z = 50%, which was observed in the range of fields of 5 (II-1s) to 8 Oe (I-2s) for different elements (Figure 16). The average sensitivity of short elements is about 15%/Oe in the range of external magnetic fields of 4 to 5 Oe. The field dependence of ΔZ/Z(H) curves are asymmetric with respect to zero magnetic field axis. This feature can be associated with magnetic hysteresis of longitudinal magnetization processes. In order to reveal all MI hysteresis, features with both branches of the MI curves should be measured in increasing external magnetic field, starting with a high negative magnetic field sufficient for magnetic saturation, and in a high positive decreasing magnetic field, also starting with the field sufficient for saturation.

Careful measurements of both MI branches allow us to evaluate even very delicate changes in the impedance variations related to magnetization processes. For example, one can see a small jump on the ΔZ/Z(H) curves observed in the region of the external fields about ±1 Oe (both in the ascending (from −100 Oe to 100 Oe) and in the descending (from 100 Oe to −100 Oe) fields (Figure 16c). Hysteresis of this type were previously described in the literature for different kinds of magnetoimpedance materials, including MI multilayered structures [63,64,65,66]. It is also consistent with the type of magnetic domains observed in the remnant state of the elements (Figure 6 and Figure 8). Interestingly, element II-1s has a non-zero MI ratio near the zero field, as well as a high sensitivity of 30%/Oe in the range of 2.5 to 3.5 Oe, which is quite suitable for electronic applications [67,68,69,70].

Magnetic hysteresis is, in general terms, a kind of research instrument used for better understanding the magnetization processes in magnetic materials. However, it is considered an undesirable feature when referring to technological applications. Even so, a real electronic device is not expected to work in a large field interval such as from +100 to −100 Oe and vice versa. If one takes in account the MI behavior corresponding to a minor loop in the range of 2.5 to 3.5 Oe, then the magnetic hysteresis can be neglected. There is also another possibility, namely the usage of the bridge configuration for a number of elements in order to minimize magnetic hysteresis. The field dependences ΔZ/Z(H) are characterized by two peaks in magnetic fields roughly symmetrical with respect to the ΔZ/Z(H = 0) axis. These peaks are located near magnetic fields close to the effective magnetic anisotropy field, which is typical for elements with the contribution of transversely induced magnetic anisotropy [2,5,9,24,66]. This result once again confirms the interpretation of the whole set of magnetic measurements.

### 3.5. Discussion and Future Trends

Keeping in mind the possibility of a massive production of rectangular FeNi/Cu-based multilayered MI elements for different kinds of magnetic field detectors, we designed a very simple structure for rf-sputtering deposition with only two sputtering targets. Sensitive elements were afterward simultaneously obtained by standard lift-off lithographic process and categorized as batches of six long and six short elements. Although the dynamic characterization methods for thin films and multilayered structures (ferromagnetic and spin-wave resonances, magnetoimpedance effect) are widely available nowadays, it is still not easy to have them all in one laboratory, especially those which focus only on the industrial development. The measurements of ferromagnetic and spin-wave resonances carried out in different orientations were useful for the definition of the angular ranges of the orientation of the applied magnetic field, within which the multilayered system of MI element can be viewed as an effective medium with a small dispersion of average parameters.

Within the many years of thin-film MI materials development, the copious research conducted were making efforts to reach the theoretical limits of the MI effect and to understand why, in practice, real materials show MI values down to two orders of magnitude lower, in comparison with theoretical limits [71,72]. Here, we observed that the field and frequency dependences of the MI ratio for different MI elements were close to each other, indicating a usefulness of the lithography method for the fabrication of a large number of elements with good repeatability of the elements’ properties in one batch. However, the elements of different parts had some distinctive features, first of all, due to the geometrical features of the borders. MI features obtained for short samples showed much closer results for the maximum value of MI ratio, MI sensitivity, and the value of the work interval (the field interval of the linear dependence of ΔZ/Z in the region of low magnetic fields). However, other contributions were not excluded.

At the same time, careful ferromagnetic resonance (FMR) and spin-wave resonance analyses indicated that, although the system can be viewed as an effective medium with a small dispersion of average parameters, the dispersion itself in such a complex layered structure should be considered as something natural and inevitable. The observed values of the MI effect are sufficient for designing small magnetic field sensor prototypes.

## 4. Conclusions

[Cu(3 nm)/FeNi(100 nm)]_5_/Cu(150 nm)/FeNi(3 nm)/Cu(150 nm)/FeNi(3 nm)/Cu(150 nm)/[FeNi(100 nm)/Cu(3 nm)]_4_/FeNi(100 nm) rectangular multilayered elements were obtained by rf-sputtering and standard lift-off lithographic processes as batches of six long (0.5 × 10.0 (mm^2^)) and six short (0.5 × 5.0 (mm^2^)) elements obtained in an identical set of steps. Their static and dynamic magnetic properties were studied. Ferromagnetic and spin-wave resonances and magnetoimpedance were used to analyze their magnetic properties at in-plane and out-of-plane orientations in order to detect the angular ranges of the orientation of the applied magnetic field within which the system can be considered as an effective medium with a small dispersion of average parameters. The analysis of the angular dependences provided a number of fundamental parameters—the effective magnetization, the exchange interaction constant, the surface anisotropy constant, and the perpendicular anisotropy field. Comparison magnetic properties of the elements having the same geometry and prepared either simultaneously or using the same procedure allowed the statistical evaluation of the functional properties.

The maximum MI values at different frequencies for the long elements are approximately doubly higher in comparison with ΔZ/Z_max_ for the short elements. The field dependences of the maximum value of MI ratio for l elements show a certain degree of difference from sample to sample with the minimum of about 93% and maximum ΔZ/Z ratio of about 105%. The absolute maximum value of 105% was observed in the external magnetic field close to the value of the magnetic anisotropy field of 7 Oe. The maximum sensitivity of 30%/Oe in the range of the external fields of 3 Oe to 5 Oe is relatively satisfactory for different applications.

## Figures and Tables

**Table 1:** Demagnetizing field of MI rectangle stripe elements $H_{d}$ calculated using Equation (1), saturation field $H_{s}$ estimated in the way shown in Figure 9.

Sample	Transverse to Stripe Element		Along the Stripe Element	
	$H_{d}$, Oe	$H_{s}$, Oe	$H_{d}$, Oe	$H_{s}$, Oe
l	35.1 ± 0.1	25 ± 1	1.7 ± 0.1	5.5 ± 0.1
s	34.9 ± 0.1	25 ± 1	3.4 ± 0.1	6.0 ± 0.1

**Table 2:** The values of the anisotropy field *H_a_*.

$M_{eff}$, G	The Perpendicular Anisotropy Field of the Individual Mode *H_a_*, Oe			
	1	2	3	4
820	120 ÷ 420	200 ÷ 1000	(750 ÷ 1240)	4500 ÷ 5000

**Table 3:** Selected parameters of the spin-wave stiffness and surface anisotropy for microwave spectra shown in Figure 14.

Area	${\tilde{\eta }}_{eff}$, Oe	$\left|K_{S1}\right|,\;e\mathrm{rg}/{\mathrm{cm}}^{2}$	$\left|K_{S2}\right|,\;e\mathrm{rg}/{\mathrm{cm}}^{2}$
A	5.0 ± 0.5	0.017 ± 0.002	0.014 ± 0.002
B	1.5 ± 0.1	0.035 ± 0.004	0.042 ± 0.004
C	9.0 ± 0.9	-	-

## Data Availability

Data available from the corresponding author upon reasonable request.
